# Stress and perceived stigma among parents of children with epilepsy

**DOI:** 10.1007/s10072-019-03822-6

**Published:** 2019-03-22

**Authors:** Akanksha Rani, Priya Teresa Thomas

**Affiliations:** Department of Psychiatric Social Work, National Institute of Mental Health and Neurosciences, Govindswamy Building (2nd Floor), Hosur Road, Near Bangalore Milk Dairy, Landmark: SBI NIMHANS Branch, Bangalore, 560 029 India

**Keywords:** Children, Epilepsy, Clinical profile, Stigma, Parental stress, Tertiary hospital

## Abstract

**Purpose:**

The present study aimed at understanding the stress and perceived stigma among parents of children with epilepsy seeking treatment at a tertiary referral center for neurology in South India.

**Materials and methods:**

Parents of sixty children suffering from epilepsy in the age group of 4–15 years were interviewed to explore parental stress and perceived stigma. They were recruited consecutively over a period of 6 months in 2015. Tools administered were Childhood-Illness related Parenting Stress Inventory (Manford in J Neurol 264(8):1811–24, 2017) and the Parent Stigma Scale (Baca et al. in Value Health 13(6):778–786, 2010).

**Results:**

The mean age of parents was 37.2 years, and the majority of parents who used to bring their child to the hospital were male (71.7%) and educated up to the secondary/intermediate level (36%) and were from lower socio-economic status. The mean age of children with epilepsy was 8.4 years with the majority of them being male (66.7%), affected with chronic seizures (58.3%) with most commonly occurring seizure type being generalized seizures (50%), with a co-morbid diagnosis of cerebral palsy (26.7%). A significant number of parents reported difficulty in communicating with medical team (58.3%) and significant others (51.7%) about their child’s seizures and difficulty in making decisions related to their child’s medical care (43.3%) which strained their financial resources and created difficulty in adequate role functioning. Findings indicated that most of the parents of children with chronic seizures perceived reactions of others to be negative (53.3%) and would limit family social interaction which resulted into emotional reaction in the form of anger, guilt, fear, anxiety, and depression.

**Conclusion:**

Parents are important figures in the process by which children with epilepsy came to acknowledge themselves being different from other children. Parents often feared divulging their child’s epilepsy to their friends and relatives because they experienced a sense of shame, self-blame, and rejection which also increased their stress.

## Introduction

Epilepsy is a common neurological disorder of childhood which has complex ramifications. Defining epilepsy can be quite problematic as it is characterized by seizures and epilepsy-like febrile seizures and drug-induced seizures [1]. Children with epilepsy because of seizures have other co-existing health conditions that can significantly affect a child’s physical health as well as psychological and social well-being.

Parental stress can be defined as the psychological and physiological reactions of the parents as they attempt to meet the challenges of caring for their sick child. Raising a child with epilepsy involved an often state of uncertainty, apprehension, and need for continued surveillance. Parents need to learn to cope with special diets, medication, schooling challenges, repeated hospitalizations, behavioral problems, and much more [2]. Diagnosis of epilepsy in a child brought with it a series of consequences for the family, and most parents got affected by it: the “loss of a perfect child” and the realization that the child might always be different from other children because of their illness [3].

Perceived stigma may have two different components: the shame associated with having epilepsy based on a sense of being not able to have control over the child’s seizures and the fear of encountering enacted stigma which may cause a parent to take efforts to hide his or her child’s health condition [4]. A negative attitude of the general public towards a person with epilepsy led to a belief that epilepsy was a disease affecting biological, cognitive, emotional, and social ability resulting into a person with epilepsy being treated differently by society even though their seizures are well controlled [5]. The structural causes like poverty, unemployment, homelessness, and violence may act as risk factors which further aggravate parental stress and stigma related to epilepsy [6, 7].

## Objective of the study

We aimed to understand the stress and perceived stigma among parents of children with epilepsy and to find out the association between parental stress and perceived stigma.

## Material and methods

A cross-sectional descriptive study was conducted in the outpatient consultation in the neurology department of a tertiary referral center in South India. Parents of 60 children who met exclusion and inclusion criteria were recruited through convenient sampling. Participants in the study were 18 years of age or older who had a child in the age-group 4–15 years affected with generalized or partial seizures. Parents of children with a co-morbid diagnosis of ADHD, autism, intellectual developmental disorder/mental retardation, and cerebral palsy were also included. Children who have been diagnosed with non-epileptic seizures, febrile seizures, and neurodegenerative disease of infancy and childhood or any other medical or psychiatric illness were excluded.

### Measures

Socio-demographic profiles of the child and parents were assessed using a self-designed performa in the form of a semi-structured interview schedule. It consisted of background information about the children, parents, and clinical profile of children. Stress was assessed using the Childhood Illness-related Parenting Stress Inventory [5]. It consists of four domains: communication, emotional functioning, medical care, and role functioning. The total score comprised of the sum for each of the four domains. The Parent Stigma Scale [6] was used to assess the stigma. It shows parental perception about how others form an opinion and view child because of epilepsy. It measures confidence in seizure management, worry, mood, and family life/leisure. Parents were asked to respond on a 5-point scale. A higher score reflects greater perceptions of stigma associated with their child having epilepsy and vice versa.

Ethical approval to conduct the study was taken from an institutional ethics committee. A written informed consent was taken from all parents prior to participating in the study.

### Procedure

Parents who came along with a child with epilepsy for outpatient consultation in the neurology department of a tertiary referral center in South India since July 2015 to December 2015 were recruited. Children were diagnosed with epilepsy by two independent neurologists and have been coming regularly for follow-up since year 1. Parents who fit into the inclusion-exclusion criteria were contacted and explained the nature of the study, confidentiality, and their right to withdraw. The parents were divided into two groups, i.e., parents of children with epilepsy with co-morbid condition and parents of children with epilepsy without co-morbid condition. The parents’ written consent was taken before they participated in the study. For parents who were not literate, the researcher read out the questions and marked the answers.

The researcher then spent some time with the child. Initial rapport had to be built with the child by engaging the child in coloring tasks or by giving a puzzle book to solve so that parents can be interviewed. Appropriate psycho-social intervention was provided post-assessment.

### Data analysis

Statistical analysis was carried using R software. The data from the questionnaires were analyzed using a descriptive statistic like (frequency and percentage) mean and standard deviation, and non-parametric tests like Mann-Whitney *U* test were done.

## Results

### Socio-demographic profile of children with epilepsy

The age range of children was 6 to 10 years with a mean age of 8.4 years. The majority of children (66.7%) affected with epilepsy were male with only 33.3% of females affected with epilepsy. A large number of children (33.3%) had not yet started going to school or dropped out after the onset of seizures (Table 1).

**Table 1:** Socio-demographic profile of children with epilepsy

Variables	Category	Frequency and percentage
Age	Less than 6 yrs.	14 (23.3%)
	Between 6 and 10 yrs.	24 (40%)
	Greater than 10 yrs.	22 (36.7%)
Gender	Male	40 (66.7%)
	Female	20 (33.3%)
Schooling	Not yet started/dropped out	20 (33.3%)
	Going to school	40 (66.7%)

### Socio-demographic profile of parents

Sixty parents have been recruited for the study. The age range of parents was 25 to 35 years with a mean age of 37.2 years. The majority of parents were male (71.7%) and educated up to the secondary/intermediate level (36%) and was doing a semi-skilled job (43.3%). Most of the parents (66.7%) were from lower socio-economic status with only 3.3% from higher socio-economic status. An average number of children came from a nuclear family (55.0%). For a maximum number of children (85.0%), their mother was the primary caregiver. There was no consanguinity among the majority of parents (78.3%) with only 21.7% reporting consanguinity predominantly third-degree relative (Table 2).

**Table 2:** Socio-demographic profile of parents

Variables	Category	Frequency and percentage
Age	25–35 years	26 (43.3%)
	36–45 years	22 (36.7%)
	46–55 years	12 (20%)
Gender	Father	43 (71.7%)
	Mother	17 (28.3%)
Education	Illiterate	5 (8.3%)
	Primary/secondary	15 (25%)
	Higher secondary/intermediate	22 (36%)
	Graduate/postgraduate	18 (30%)
Occupation	Unemployed/homemaker	11 (18.3%)
	Unskilled	8 (13.3%)
	Semi-skilled	26 (43.3%)
	Skilled	15 (25.0%)
Economic status	Lower	40 (66.7%)
	Middle	18 (30.0%)
	Upper	2 (3.3%)
Type of family	Nuclear	33 (55.0%)
	Joint	27 (45.0%)
Primary caregiver	Father	5 (8.3%)
	Mother	51 (85.0%)
	Others	4 (6.7%)
Consanguinity among parents	No	47 (78.3%)
	Yes	13 (21.7%)

### Clinical profile of children

Clinical details of children were assessed by systemically reviewing case files and treatment details. The result significantly indicated that 58.3% of children have cases of chronic seizures whereas 41.7% have cases of new-onset seizures. Although the chronic sample included children with seizures that had begun as early as birth, the new-onset sample limited the lowest age of onset to 4 years. The findings also show that an average number of children (50%) had generalized seizures with only 31.7% of children having partial seizures followed by 18.3% of children having a combination of both generalized and partial seizures. In terms of frequency of seizure episodes, a significant number of children (45%) had episodes of seizures less than ten times a day. Most of the children had seizures less than 5 s (63.3%). The majority of children (73.3%) seizures have not been controlled. In terms of co-morbid conditions, 26.7% of children had cerebral palsy followed by 16.7% of children having an intellectual developmental delay with only 3.3% of children having autism and attention deficit hyperactivity disorder. When it comes to other associated problems along with seizures, many children (31.7%) had memory problems followed by 23.3% having difficulty in speech, temper tantrums, and anger outburst (Table 3).

**Table 3:** Clinical details of children

Variables	Category	Frequency and percentage
Diagnosis of seizure disorder	Chronic	35 (58.3%)
	New onset	25 (41.7%)
Main seizure type	Generalized seizures	30 (50.0%)
	Partial seizures	19 (31.7%)
	Combination of both generalized and partial	11 (18.3%)
Duration of seizure	Less than 5 s.	38 (63.3%)
	Between 5 and 10 s.	17 (28.3%)
	Greater than 10 s.	5 (8.3%)
Frequency of episodes	Less than 10 times a day	27 (45.0%)
	Between 10 and 20 times a day	15 (25.0%)
	More than 20 times a day	18 (30.0%)
Seizure control	Not control	44 (73.3%)
	Control	16 (26.7%)
Co-morbid condition	No co-morbidity	30 (50.0%)
	Cerebral palsy	16 (26.7%)
	Intellectual developmental delay	10 (16.7%)
	Autism	2 (3.3%)
	Attention deficit hyperactivity disorder (ADHD)	2 (3.3%)
Associated problems	No problem	8 (13.3%)
	Difficulty in speech	14 (23.3%)
	Temper tantrums and anger	14 (23.3%)
	Memory problems	19 (31.7%)
	Combination of any two	5 (8.3%)

### Parental stress

Parenting stress was assessed using the Childhood Illness-related Parenting Stress Inventory [5]. In communication domain, most of the parents felt confused (63.3%) about the information given to them about their child’s illness and found it difficult to speak with doctors (58.3%). The majority of the parents felt misunderstood (55%) and had arguments (51.7%) within and outside the family. An average number of parents worried about their child’s illness and found it difficult to speak to the nurse.

In medical care domain, most of the parents find it difficult to bring the child to the clinic for the treatment and had difficulty in attending to the child’s hygiene needs (48.3%). Many parents felt sad and worried to see their child having trouble eating (45%). A large number of parents had difficulty in taking decisions related to their child’s medical care (43.3%). For being with the child during medical care and handling changes in medicines and treatment, the majority of the parents had difficulty (41.7%).

In emotional distance domain, an average number of parents felt isolated. The majority of parents had frequent mood changes, felt numb inside and helpless, and had mood worsen on learning upsetting news (46.7%). Most of the parents were worried about the impact of seizures, and their mood worsens on knowing the child is in pain or getting hurt due to seizure episodes (43.3%).

In role function domain, an average number of the parents reported significant changes in their relationship with the spouse; spending more time in an unfamiliar setting like hospitals, clinics, and lab; and missing important events in their life (51.7%). The majority of the parents find it difficult to attend to the needs of other family members (48.3%). Most of the parents found it difficult and uncertain to discipline their sick child and had little time for their own needs (46.7%). A large number of parents were unable to go to work regularly (45%) (Table 4).

**Table 4:** Parental stress

Variables	Category	Frequency and percentage
Communication	Arguing	31 (51.7%)
	Speaking with doctor	35 (58.3%)
	Feeling confused	38 (63.3%)
	Talking with the nurse	30 (50%)
	Disagreeing	21 (35%)
	Feeling misunderstood	33 (55%)
	Speaking with child	28 (46.7%)
	Speaking with family	29 (48.3%)
	Worrying	30 (50%)
Medical care	Bringing my child to the clinic	29 (48.3%)
	Watching trouble eating	27 (45%)
	Being with my child	25 (41.7%)
	Making decisions	26 (43.3%)
	Helping/hygiene needs	29 (48.3%)
	Handling changes	25 (41.7%)
	Helping/procedures	24 (40%)
	Watching/procedures	24 (40%)
Emotional distance	Difficulty sleeping	27 (45%)
	Learning upsetting news	28 (46.7%)
	Seeing mood changes	28 (46.7%)
	Waiting for test results	22 (36.7%)
	Family difficulties	22 (36.7%)
	Knowing/hurting	26 (43.3%)
	Seeing child sad	28 (46.7%)
	Thinking about/isolated	30 (50%)
	Feeling numb inside	28 (46.7%)
	Worrying about the impact	26 (43.3%)
	Feeling helpless	28 (46.7%)
	Feeling uncertain	24 (40%)
	Other children seriously ill	24 (40%)
	Heart beats fast	22 (36.7%)
	Child condition getting worse	25 (41.7%)
Role function	Being unable to go to work	27 (45%)
	financial trouble	22 (36.7%)
	Attend to needs of family members	29 (48.3%)
	Being far away from family	22 (36.7%)
	Little time for my own needs	28 (46.7%)
	Being in the hospital	23 (38.3%)
	Uncertainty in disciplining my child	28 (46.7%)
	Missing important events	31 (51.7%)
	Change in relationship with the spouse	31 (51.7%)
	Spending time in unfamiliar settings	31 (51.7%)

### Perceived stigma among parents of children with epilepsy

Perceived stigma was assessed by using the Parent Stigma Scale [6]. An average number of parents felt that their child was being labeled or stigmatized due to having frequent and active seizures (53.3%). Majority of the parents reported that their child was given differential treatment because of having frequent episodes of seizures. Most of the parents worried about finding prospect groom or bride for their sick child (41.7%). Many parents reported that people have perceived notions about their child’s seizures (36.7%) and that their child has to always prove him/herself because of seizures (35%) (Table 5).

**Table 5:** Stigma perceived by parents of children with epilepsy

Category	Frequency & Percentage
Differential treatment because of the child’s seizures	28 (46.7%)
People having a notion about child’s seizures	22 (36.7%)
Child has to always prove him/herself because of seizures	21 (35%)
Difficult to find prospect groom or bride	25 (41.7%)
Child being labeled or stigmatized due to seizures	32 (53.3%)

### Assessing stress and perceived stigma among parents of children with epilepsy

The table shows that parents of children with epilepsy and co-morbid condition exhibit greater frequency of parental stress (±130.50) making it more difficult for them to cope up and manage their stress and have a higher perceived stigma (±21.90) compared with parents of children with epilepsy without co-morbid condition (Table 6).

**Table 6:** Stress and perceived stigma among parents of children with epilepsy

Study variables	Parents of children with epilepsy and co-morbid condition (*n* = 30)	Parents of children with epilepsy without co-morbid condition (*n* = 30)
	(mean±SD)	(mean ±SD)
Parental stress (frequency)	130.50 ±24.483	110.43 ±25.923
Parental stress, (difficulty)	131.47 ±24.554	111.40 ±25.370
Perceived stigma	21.90 ± 3.800	19.60 ± 2.111

### Comparison of stress and perceived stigma among parents of children with epilepsy

The table indicates that the frequency of parental stress (*U* = 278, *ρ* = .011) is significantly higher increasing difficulty to cope with stress (*U* = 275, *ρ* = .010) for the parents of children with epilepsy and co-morbid condition as compared with those of the parents of children with epilepsy without co-morbid conditions. The results significantly indicated that parents of children with epilepsy and co-morbid conditions have a higher perceived stigma (*U* = 243, *ρ* = .002) as compared with parents of children with epilepsy without co-morbid condition (Table 7).

**Table 7:** Comparison of stress and perceived stigma among parents of children with epilepsy

Study variables	Parents of children with epilepsy and co-morbid condition (*n* = 30) (mean rank)	Parents of children with epilepsy without co-morbid condition (*n* = 30) (mean rank)	Mann-Whitney *U* test	*p* value
Parental stress, frequency	36.22	24.78	278.500	.011
Parental stress—difficulty	36.32	24.68	275.500	.010
Perceived stigma	37.38	23.62	243.500	.002

## Discussion

The current study was an attempt to understand the stress among parents of children with epilepsy. Some of the demographic factors associated with high parenting stress were young parental age, lower education status, and lower socio-economic status. Multiple studies have shown that if parents are less educated and have financial instability then they spent most of their income, time, and effort on child’s treatment and care. This results in exhaustion of existing economic and social resources which negatively affects a parent’s quality of life [7, 8].

The current research showed that parents of children whose seizures are not well controlled reported more stress. One of the previous studies highlighted that the seizures when poorly controlled may be disabling and interfere with the child’s ability to learn, grow, and develop normally [9]. Most of the children in the present study who had co-morbid conditions like cerebral palsy and intellectual developmental delay needed supervision and assistance in activities of daily living like feeding, bathing, taking medicines, communication, and mobility, thus increasing physical and emotional dependence on parents which resulted into a high level of parental stress. One of the studies has reported that apart from the physical dependency of the child on parents there were secondary factors such as myths and misconceptions about epilepsy, enacted stigma, and lack of knowledge of families about epilepsy directly related to parental stress and quality of care provided to the child [10, 11].

The majority of children in this study has either not yet started going to school or dropped out after the onset of seizures. These were those children for whom seizure started in quite a young age mostly when they were infant which affected their socio-emotional and cognitive development. Parents also feared that their child will have an episode of seizures at school and teachers would be unable to handle it. There were also concerns about if school authority and children came to know about the child’s seizures that they will treat the child differently, doubt the child’s ability to perform well, or labeled the child to be epileptic which made the parents further isolate the child by restricting family and social activities. This finding did not appear elsewhere in the epilepsy literature, but similar findings have been categorized differently in different studies.

In the present study, the majority of parents reported that friends and relatives who knew that the child had epilepsy treated the child differently in terms of feeling uncomfortable to be left alone with the child or considering the child not as intelligent as children of his age group. Jacoby and Austin [12] highlighted that friends or relatives would feel nervous around a child with epilepsy and would become afraid to be left alone with the child as they did not know how to perform first aid if the child had an episode of seizure. Studies have indicated that when seizures occur quite early in life and the person is being frequently quiet and resistant to treatment then the person is at higher risk of cognitive deficits which could also depend upon other factors like the number, duration, type of seizure, and antiepileptic drug therapy [13, 14].

Most of the time, mothers undertake the job of nursing the sick child and fathers played an assistive role. Etemadifar and colleague [15] reported that the majority of caregivers for patients with epilepsy are female housekeepers who care for many hours daily which significantly increase their levels of stress, anxiety, and depression. Mothers always had to be highly alert and vigilant because of the uncertainty of where and how their child will get seizures which made them over-protective and over-concerned about the child’s health and well-being. They were also described to be permissive and uncertain about disciplining the child or excessive restrictive towards the child in non-health domains like participating in sports activities or not allowing the child to move around freely in the neighborhood. One of the study concluded that the fear of a child having an episode of seizures when parents are not around and sense of helplessness on seeing the child in pain made parents to be permissive or exert control and restrictions in their child’s day to day life which often occurred for longer period of time than what can be considered reasonable or appropriate [16].

Parents felt helpless and sad on seeing the child’s life being adversely affected by epilepsy; as the severity of seizures increased, parents became more desperate to find a cure for their child’s illness. The more desperate they became, the worst they felt. Parents reported that worrying would give them mental peace and help them deal with feelings of guilt and failure as a parent. The excessive worrying often resulted in negative emotions like fear, anxiety, and sadness. Jensen and colleague [9] concluded that parents kept worrying about their child’s health which could deteriorate at any time which affected parents’ physical and mental health leading to sleep deprivation, easy fatigability and feeling of helplessness, despair, and anger.

Disease management issues were frequently reported by parents in the current study in the form of bringing the child to the hospital for consultation; taking decisions related to medical tests and change in medication, medicine supervision, managing side-effects of medication; and being with the child throughout the treatment procedure at the hospital. Streisand [5] found that parents caring for children with a debilitating illness like epilepsy are at greater risk of having stress and increase level of stress could negatively affect the quality of care provided to the child.

In the current study, most of the parents had difficulty in terms of discussing with the doctor about child’s seizure condition, feeling confused about the information provided to them because of technical terms and medical jargons used by the doctors. Parents felt quite hesitant to discuss about their child’s illness with relatives and friends or affected child as they did not know what to say and how to say so they kept worrying about it. Hobdell and colleague [17] found that parents’ inability to effectively manage child’s seizure condition which was due to lack of adequate information and skills took an emotional toll on family members by increasing their worries and concern leading to negative emotions like anxiety, despair, helplessness, and sadness.

Most of the parents were missing important events in their life and has made their child as the center of their attention. As a result, they had little time to meet their own needs or spend time with family members. Camfield [4] discussed in his study that parents missed important events in their lives and socially isolated themselves because of the fear of divulging their child’s epilepsy to their friends and relatives as they experienced a sense of shame, self-blame, and rejection. Higher stigma was associated with more worry, parent negative mood, and the adverse impact of epilepsy on parent life and leisure activity [18]. Epilepsy literature suggests that parents’ quality of life deteriorates after the onset of a child’s illness which have an effect on their adaptation, role functioning, and coping styles [8, 19].

Most of the parents were concerned about the impact of epilepsy on their child’s future if it continues in adulthood. Some of their concerns were whether parents would be able to find a bride or groom for the child and child’s ability to conceive and perform conjugal responsibilities adequately. Previous studies have reported that most of the parents of children with chronic seizures had concerns regarding child’s marriage especially concerns regarding the ability to conceive, fear of disclosure about epilepsy before marriage, and consequences of disclosure. They perceived reactions of others to be negative, and this belief was shaped by seeing the general public’s negative attitude towards a person with epilepsy [20, 21].

One of the factors which played an important role in helping the child to adjust to epilepsy was a parental reaction to the child’s diagnosis which set the stage for their child’s own interpretation of its significance. If parents’ reactions were negative, the child learnt to think about epilepsy as something they should be ashamed of which resulted into social isolation [22, 23]. Child’s extended family members, neighborhood friends, and teachers acted what Hintermair [23] called as “stigma coaches” which was positively associated with a behavior problem and socio-emotional problems in their children [24].

Some of the constraints in the generalization of findings are small sample size, cross-sectional nature of the study, time constraint, and sampling bias. The acceptance of the parents’ emotional reactions of grief, anger, fear, or guilt is essential to facilitate parents’ coming to terms with their child’s illness. Parents also need the emotional support of treating team when having to negotiate appropriate restrictions on their child’s activities and when being faced with difficult decisions such as whether a change in medication or new treatment options, e.g., surgical treatment which to them may seem terrifying, should be explored.

## Conclusion

Parental reaction to the child’s diagnosis set the stage for the child’s own interpretation of its significance. As the child grew older, parental stress was likely to increase due to management difficulties, financial strains, and increased concern about the child’s future. The addition of behavioral problems, a common occurrence in adolescents due to hormonal changes and side-effects of antiepileptic medication, further increased stress and burden of parents, thus increasing stigma related to epilepsy. Treating team can be sensitive to these needs and spend some time with the parents before making any treatment-related changes, and attention can be given to parent’s involvement in the child’s management.
